# *Berberis aristata*, *Elaeis guineensis* and *Coffea canephora* Extracts Modulate the Insulin Receptor Expression and Improve Hepatic Steatosis in NAFLD Patients: A Pilot Clinical Trial

**DOI:** 10.3390/nu11123070

**Published:** 2019-12-16

**Authors:** Valentina Cossiga, Vincenzo Lembo, Maria Guarino, Concetta Tuccillo, Federica Morando, Giuseppina Pontillo, Andrea Fiorentino, Nicola Caporaso, Filomena Morisco

**Affiliations:** 1 Gastroenterology Unit, Department of Clinical Medicine and Surgery, University of Naples “Federico II”, 80131 Naples, Italy; valentina.cossiga@gmail.com (V.C.); v.lembo@hotmail.it (V.L.); maria.guarino86@gmail.com (M.G.); federicamorando.fm@gmail.com (F.M.); giu.pontillo@gmail.com (G.P.); andreapraiano@gmail.com (A.F.); nicola.caporaso@unina.it (N.C.); 2 Department of Clinical and Experimental Medicine, University of Campania “Luigi Vanvitelli”, 80131 Naples, Italy; concetta.tuccillo@unicampania.it

**Keywords:** NAFLD, insulin receptor, hepatic steatosis, controlled attenuation parameter (CAP)

## Abstract

Non-alcoholic fatty liver disease (NAFLD) is associated with insulin resistance and diabetes. A reduction in insulin receptor (IR) expression has been reported in these patients. The aims of this study were to evaluate the effects of a mixture of plant extracts consisting of *Berberis aristata*, *Elaeis guineensis* and decaffeinated green coffee by *Coffea canephora* on the improvement of glycaemic profile, through the modulation of IR levels, and of hepatic steatosis in NAFLD patients. Forty-nine patients with a grade of steatosis S1-S2 were randomly allocated to the treatment with plant extracts or placebo for six months. Hepatic steatosis was evaluated using transient elastography with CAP (controlled attenuation parameter). Glucose, insulin, and IR levels were measured in serum samples. At the end of the study, patients treated with plant extracts displayed a significant reduction of serum glucose (*p* < 0.001), insulin levels (*p* < 0.01), homeostatic model assessment for insulin resistance (HOMA-IR) index (*p* < 0.001), and CAP value (*p* < 0.01) compared to placebo. Moreover, the IR expression was increased significantly in the plant extracts group compared to the placebo group (*p* < 0.05). The combination of plant extracts increases serum IR levels, determining amelioration of glycemic profile and improvement of hepatic steatosis in NAFLD patients.

## 1. Introduction

Non-alcoholic fatty liver disease (NAFLD) is a growing cause of chronic liver disease globally [1]. It is characterized by excessive fat deposition in the liver and it is often associated with obesity, insulin resistance, and features in metabolic syndrome. Generally, individuals with insulin resistance have decreased levels or absence of Insulin Receptor (IR) expression [2]. The IR is a membrane-spanning glycoprotein that is essential for the action of insulin. Binding of insulin to IR in the liver, muscles, or adipose tissue triggers multiple intracellular pathways that cause a reduction of blood glucose levels [3,4].

Recently, the prevalence of NAFLD has increased worldwide as a result of the epidemics of type 2 diabetes mellitus and obesity, and in Europe it is about 25% [5]. The progression of NAFLD can lead to non-alcoholic steatohepatitis (NASH) that shows a potentially progressive path to liver fibrosis, cirrhosis, and hepatocellular carcinoma. All these complications can pose significant health and economic problems [6].

Thus, it is critical to identify patients at higher risk of NASH and advanced fibrosis to optimize their management. The gold standard to diagnose NASH and liver fibrosis is a liver biopsy, but its invasive nature, high cost, and inter- and intra-observer variability make it unsuitable for screening at a population level [7]. Therefore, presently, it is common to assess liver fibrosis using liver stiffness measurement (LSM) by transient elastography. However, the diagnostic performance of LSM is strongly affected by obesity and severity of steatosis that may increase false-positive rates [8].

Notably, in the last few years, a new software of the FibroScan machine was available to quantify hepatic steatosis, the so-called controlled attenuation parameter (CAP) value, which can be used in patients with NAFLD to avoid overestimation of liver fibrosis [9].

Currently there are no approved drugs for the treatment of NAFLD and lifestyle modification is the only approved treatment. However, some plant extracts are used as adjuvants to conventional therapies to improve hepatic steatosis in NAFLD patients [10].

Previous experimental studies suggest that berberine, a plant alkaloid found in *Berberis aristata*, has hypoglycemic and insulin-sensitizing activity both in animal models and in type 2 diabetes mellitus patients through the modulation of insulin receptor and hypocholesterolemic activity through the modulation of the LDL receptor [11]. Moreover tocotrienols, components in vitamin E found in *Elaeis guineensis*, reduce cholesterol and triglyceride levels through the modulation of lipogenic genes and reduce the oxidative damage directly by exerting their antioxidant activity. Reduced oxidative stress can improve insulin sensitivity, which in turn might decrease plasma triglycerides and free fatty acid through down-regulation of lipolysis [12]. Additionally, experimental studies also showed that coffee consumption shows antioxidant properties through modulation of gene expression of some inflammatory proteins [13].

Therefore, the aim of this study was to evaluate the effects of a combination of plant extracts consisting of *Berberis aristata*, *Elaeis guineensis* and decaffeinated green coffee by *Coffea canephora*, (Trixy^®^, Nathura S.p.A., Montecchio nell’Emilia, Italy) on the glycemic profile and on the improvement of hepatic steatosis of NAFLD patients. Based on the scientific evidence of the plant extracts effects on glycemic profile, this combination was used for evaluate their synergistic effect on insulin resistance and in particular on hepatic steatosis.

## 2. Materials and Methods

### 2.1. Study Design

Studied patients were selected from adults referred to the Gastroenterology and Hepatology Unit of the University of Naples “Federico II” (Naples, Italy) for whom a diagnosis of NAFLD (grades 1–2) was made according to biochemical and instrumental data and significant liver fat accumulation, defined as a value ≥214 dB/m using controlled attenuation parameter (CAP) during transient elastography.

Exclusion criteria included pregnancy or breastfeeding, excessive alcohol intake (men >30 g/day, women >20 g/day), body mass index (BMI) ≥35 Kg/m^2^, consumption of hypoglycemic and hypolipidemic medications, vitamin E, as well as any drug known to affect hepatic function, and presence of hepatitis, coronary, renal, pulmonary, and thyroid diseases.

Moreover, during the study, patients did not have to change their lifestyle, maintaining stable body weight (not varying >10%) and their pharmacological therapy.

A controlled clinical trial was conducted on forty-nine patients, randomly (via alternative assignment to tablets bottles coded as B or C) allocated to the plant extracts (*n* = 26) or placebo (*n* = 23) group (1 tablet per day for six months).

The plant extracts consist of 588 mg of *Berberis aristata* (500 mg of berberine), 143 mg of *Elaeis guineensis* (30 mg of tocotrienols), and 67 mg of decaffeinated green coffee by *Coffea canephora* (30 mg of chlorogenic acid). The plant extracts were generously donated by Nathura S.p.A., Italy. The placebo, free of plant extracts, was formulated with the same composition of excipients that make up the nutraceutical.

The study protocol was approved by the Ethical Committee of the University of Naples “Federico II” (protocol number: 174/16) and all participants signed the informed consent before the enrolment.

### 2.2. Anthropometric Measurements

The anthropometric measurements included height (m), weight (Kg), body mass index (BMI, Kg/m^2^), waist circumference (cm), hip circumference (cm), and waist-hip ratio (WHR).

### 2.3. Biochemical Measurements

Serum levels of alanine aminotransferase (ALT), aspartate aminotransferase (AST), gamma-glutamyl transpeptidase (γ-GT), cholesterol, triglycerides, as well as glucose and insulin were measured after an overnight fast, at baseline, and the end of the study using calorimetric standard bio-chemical assays at the central laboratory. The homeostatic model assessment for insulin resistance (HOMA-IR) index was calculated based on fasting blood glucose and insulin levels at baseline and at end-of-study.

### 2.4. Serum Insulin Receptor Measurement

The serum insulin receptor (IR) levels were measured via enzyme-linked immunosorbent assay (ELISA) using a commercial kit (Elabscience^®^, Houston, TX, USA). Assays were carried out according to the manufacturer’s instructions.

### 2.5. Quantification of Hepatic Steatosis

Hepatic steatosis was assessed non-invasively using vibration controlled transient elastography (Fibroscan, Echosens SA, Paris, France) with controlled attenuation parameter (CAP). The CAP value is a median, it is expressed in decibel per meter (dB/m) and ranges from 100–400 dB/m. Higher values denote greater liver fat contents. The examination was performed when the patient fasted for a minimum of 5 h. Patients with a median CAP value obtained from at least 10 measurements and included in the range from 214 to 311 dB/m, corresponding to the grade of steatosis S1-S2 [14], were enrolled.

### 2.6. Statistical Analyses

Statistical analyses were performed using GraphPad Prism 5.0 (GraphPad Software Inc., San Diego, CA, USA). The data are presented as means ± standard deviations (SD), or medians with ranges. Continuous variables were compared between plant extracts and control groups using the *t*-test (for normally distributed data) or the Mann–Whitney U test, as well as the chi-square test (for non-normally distributed data). The first and second comparisons for normal or asymmetrical distributions were made by paired *t*-test or the Wilcoxon, respectively. All *p*-values < 0.05 were considered statistically significant.

## 3. Results

Forty-nine patients participated in this study and follow-up was completed by all patients at six months. The median age was 51.5 ± 10.9 years and 57 ± 12.1 years in the plant extracts and placebo group, respectively, without difference in the median age between the two groups (*p* = 0.218). Thirty-three patients were male (66%), 17 in the plant extracts and 16 in placebo group, respectively, and there was no difference in the proportion of the gender between the groups (*p* = 0.545). Clinical characteristics and metabolic features of the patients pre- and post-treatment are summarized in Table 1.

### 3.1. The Effect of Nutraceutical on Metabolic Parameters

Triglycerides, total cholesterol, HDL, and LDL cholesterol were evaluated at baseline and at the end of the study. In both groups there was not a significant improvement of these parameters (Table 1).

### 3.2. The Effect of Nutraceutical on Glycemic Profile and Insulin Resistance

At the baseline, there was no difference between the treated and placebo group for glucose levels (*p* = 0.934), insulin levels (*p* = 0.663), and HOMA-IR index (*p* = 0.697) (Table 1).

**Figure 1:**
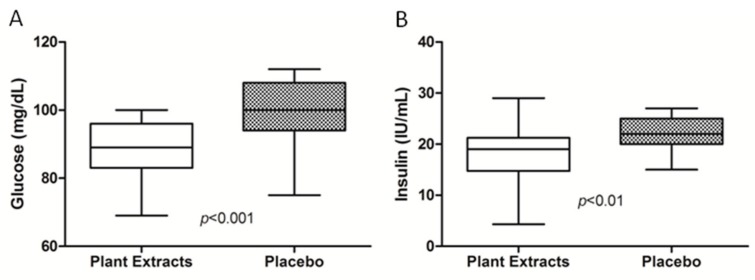
Glucose (**A**) and insulin (**B**) at end-of-study in the plant extracts and placebo group. Data are presented as mean ± SD.

Instead, at the end of study there was a significant improvement of glycemic profile. In particular, the results showed a significant reduction of glucose levels in the plant extracts group (88.75 ± 7.5 mg/dL) in comparison to the placebo group (98.8 ± 9.9 mg/dL) (*p* < 0.001) and a reduction of insulin levels between the treated (17.9 ± 5.7 IU/mL) and placebo (22.3± 3.3 IU/mL) group (*p* < 0.01) (Figure 1). As a result of the reduction of glucose and insulin levels, the HOMA-IR index showed a significant amelioration in the plant extracts compared to placebo group (*p* < 0.001) (Table 1).

**Figure 2:**
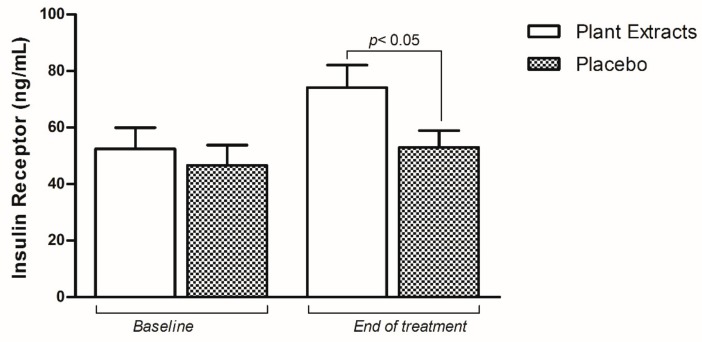
Comparison of insulin receptor (IR) levels in plant extracts and placebo group at baseline and end-of-study. Data are present as mean ± SD.

To explain the improvement of glycemic profile, the IR levels were analyzed. At the baseline of the study there was no difference between the plant extracts (52.48 ± 30.88) and placebo group (46.62 ± 29.47) (*p* = 0.575). On the contrary, at the end of study there was a significant increase of IR levels in the treated group (74.14 ± 32.77) compared to the placebo group (52.93 ± 24.94) (*p* < 0.05), that could explain the amelioration of the glycemic profile (Figure 2).

### 3.3. The Effect of Nutraceutical on Hepatic Steatosis

At the baseline, the median CAP values were 291.6 ± 39.2 dB/m and 289.1 ± 30.8 dB/m in the plant extracts and placebo group, respectively, without statistically significant difference (*p* = 0.403).

**Figure 3:**
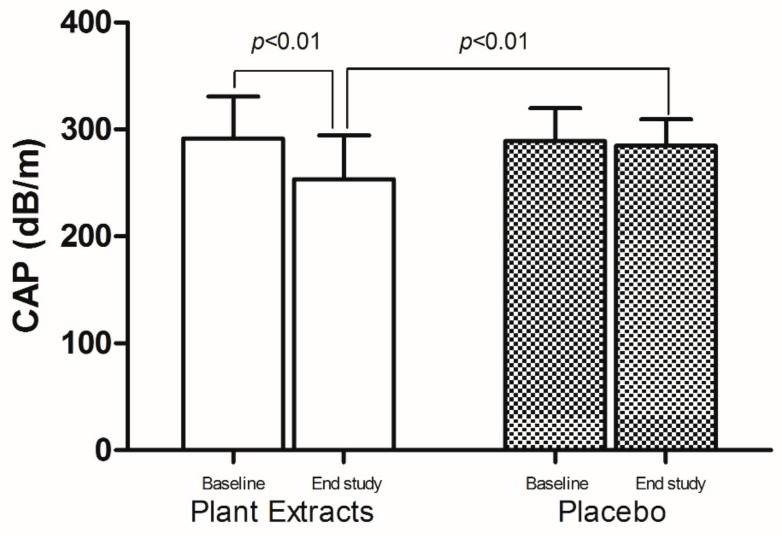
Comparison of the controlled attenuation parameter (CAP) values in the plant extracts and placebo group at baseline and end-of-study. Data are presented as median (range).

At the end of study, the median CAP values were 251.3 ± 41.5 dB/m in the treated with plant extracts group and 281.7 ± 35.3 dB/m in the placebo group (*p* < 0.01) (Figure 3). Moreover, in the treated group the reduction of CAP values, at the end of study, was significant ameliorated (*p* < 0.001) in comparison to baseline. Differently, in the placebo group there was not a significant improvement of CAP values between baseline and end of study (*p* = 0.447) (Figure 3).

## 4. Discussion

NAFLD is the most common cause of chronic liver disease worldwide [15] and is considered the hepatic manifestation of metabolic syndrome. Among the components of the metabolic syndrome, type 2 diabetes and insulin resistance seem to be the most relevant risk factors for NAFLD/NASH and clinical predictors of adverse outcomes [16].

A change of lifestyle focused on weight loss remains, at the moment, the only therapeutic approach of NAFLD, even if several studies indicate that some plant extracts, in combination with conventional therapy, improve the metabolic pattern [10]. Furthermore, previous studies analyzed the effects of berberine, tocotrienols, and decaffeinated green coffee, separately, on glucose and lipid metabolism in animals and humans [11,12,13], but the mechanism of action remains unknown.

Kong WJ et al. [11] showed that in cultured human liver cells, berberine increased both IR and LDLR (low density lipoprotein receptor) expression, determining a cellular response against insulin resistance. Vafa M. et al. [12] demonstrated in a double-blind, placebo-controlled, randomized trial that the addition of tocotrienols to antidiabetic drugs in patients with type 2 diabetes improved glucose blood levels in comparison to placebo. Finally, Vitaglione P. et al. [13] showed that the addition of decaffeinated coffee to a high fat diet (HFD) in rats determined a reduction in hepatic fat accumulation, systemic and liver oxidative stress, and liver inflammation.

Our pilot study showed that the use of a combination of *Berberis aristata*, *Elaeis guineensis* and decaffeinated green coffee by *Coffea canephora* extracts for six months increased serum IR levels, improved insulin resistance, and reduced the grade of hepatic steatosis.

At baseline, all the patients enrolled in our study showed insulin resistance and NAFLD, while, at the end of study, patients treated with plant extracts showed a significant amelioration of glycemic metabolism compared to the placebo group through the improvement of blood glucose and insulin levels with subsequent improvement of insulin resistance. Furthermore, the treatment with plant extracts induces a significant reduction of hepatic steatosis, assessed by CAP methodology.

These results seem to be associated with the modulation of serum IR levels. At baseline, no difference of IR levels was observed between the two groups; at the end of the study a significant increase of serum IR levels was observed in the treated group compared to the placebo group (*p* < 0.05). Therefore, we hypothesized that the improvement of the glycemic and insulin resistance profile could be related to a greater availability of IR on cell surface.

Previous studies reported that berberine has hypoglycemic and insulin-sensitizing activity, and several mechanisms were hypothesized. It was shown that the berberine upregulates the expression of IR suggesting an improvement of insulin signaling and resistance [11,17,18]. In particular, Kong WJ et al. hypothesized that the mechanism of action of berberine was to increase the IR expression at the transcriptional level by stimulating its promoter, with a crucial role played by the activation of a protein kinase C (PKC) [11]. In addition, Zhang H et al. demonstrated that berberine has its insulin-sensitizing effect through the activation of adenosine monophosphate-activated protein kinase (AMPK), which may play a role in reducing insulin resistance [17]. Finally, in a recent paper three mechanisms were hypothesized of berberine improvement of insulin resistance: inhibition of mitochondria function, increase the breakdown of glycogen, and activation of the AMPK signaling pathway [18].

Although it is not possible to discriminate which component of the extracts mixture has led to the improvement of the glycemic profile, it is possible to hypothesize a synergistic action of the three plant components. In some studies, it was hypothesized that tocotrienols act by restoring glucose transporter type 4 (Glut4) expression and Akt (protein kinase B) signaling in skeletal muscle of diabetic mice [19,20]. Moreover, coffee consumption is associated with higher insulin secretion, insulin sensitivity, and β-cells function [21,22].

Our study shows some limitations. Firstly, it indirectly evaluates hepatic steatosis with CAP measurements that have high reliability (>95%) and low failure (<2.5%) rates, even if its values can be affected by many factors, such as obesity and operator experience [23]. In our monocentric study, this latter limit was exceeded by a single trained operator realizing all CAP evaluations.

Secondly, our study is a small randomized clinical trial and it is necessary to increase the number of patients and to continue to investigate the effects of this combination of plant extracts.

## 5. Conclusions

In conclusion, NAFLD represents a growing cause of liver disease and it is often associated with metabolic syndrome. There is no drug approved for NAFLD, but it is common to advise lifestyle modifications and to cure the single risk factor with hypoglycemic, lipid-lowering, and anti-hypertensive agents [24]. In this context, our randomized controlled trial demonstrated that six months of supplementation with the combination of *Berberis aristata*, *Elaeis guineensis* and decaffeinated green coffee by *Coffea canephora* induces an increase of serum IR levels, with the improvement of insulin resistance and hepatic steatosis.

## Figures and Tables

**Table 1:** Clinical characteristics and metabolic features of studied patients at baseline and end-of-study.

Variable	Baseline			End-of-Study		
	Plant Extracts (*n* = 26)	Placebo (*n* = 23)	*p*-Value	Plant Extracts (*n* = 26)	Placebo (*n* = 23)	*p*-Value
BMI (Kg/m^2^)	28.6 ± 3	28.2 ± 2.8	0.633	27.8 ± 2.8	27.6 ± 3	0.756
Waist-Hip Ratio	0.93 ± 0.05	0.94 ± 0.05	0.308	0.93 ± 0.07	0.9 ± 0.07	0.405
AST (times ULN)	0.69 ± 0.3	0.60 ± 0.2	0.256	0.63 ± 0.3	0.6 ± 0.2	0.622
ALT (times ULN)	0.85 ± 0.5	0.64 ± 0.3	0.09	0.72 ± 0.3	0.7 ± 0.3	0.695
γ-GT (times ULN)	1.1 ± 1.3	1.0 ± 1.1	0.788	0.98 ± 1.1	1 ± 0.9	0.695
Triglycerides (mg/dL)	154.5 ± 43.4	142 ± 54.6	0.375	134.5 ± 59.9	143 ± 54.5	0.602
Total cholesterol (mg/dL)	194.7 ± 36	191.3 ± 19	0.681	182.9 ± 29.7	193.4 ± 23.3	0.135
HDL cholesterol (mg/dL)	42.1 ± 10.8	39.7 ± 8	0.374	44.2 ± 9.8	42 ± 8.9	0.333
LDL cholesterol (mg/dL)	120.3 ± 28.1	107.2 ± 20.5	0.07	109,5 ± 29.4	116.4 ± 20.7	0.136
Glucose (mg/dL)	97.7 ± 16.6	98 ± 11.9	0.934	88.8 ± 7.5	98.8 ± 9.9	<0.001
Insulin (IU/mL)	23.8 ± 6.5	23.1 ± 2.9	0.663	17.9 ± 5.7	22.3 ± 3.3	<0.01
HOMA-IR index	5.8 ± 2.3	5.6 ± 1.2	0.697	3.9 ± 1.3	5.5 ± 1.2	<0.001
Liver Stiffness (kPa)	6.2 ± 3	6.1 ± 3.8	0.298	5.7 ± 2.7	5.4 ± 1.3	0.763

Data are presented as mean ± SD. Statistical significance was evaluated using the Student’s *t*-test or Chi-square test. *p*-values < 0.05 were considered statistically significant. Abbreviations: AST: aspartate aminotransferase; ALT: alanine aminotransferase; GGT: gamma-glutamiltransferase; HDL: high density lipoprotein cholesterol; LDL: low density lipoprotein cholesterol; HOMA: homeostatic model assessment for insulin resistance.
